# Statistics in the Genomic Era

**DOI:** 10.3390/genes11040443

**Published:** 2020-04-18

**Authors:** Hui Jiang, Kevin He

**Affiliations:** Department of Biostatistics, University of Michigan, Ann Arbor, MI 48109, USA; kevinhe@umich.edu

In recent years, technology breakthroughs have greatly enhanced our ability to understand the complex world of molecular biology. Rapid developments in genomic profiling techniques, such as high-throughput sequencing, have brought new opportunities and challenges to the fields of computational biology and bioinformatics. Furthermore, by combining genomic profiling techniques with other experimental techniques, many powerful approaches (e.g., RNA-Seq, Chips-Seq, single-cell assays, and Hi-C) have been developed in order to help explore the complex biological systems. As more genomic datasets become available, both in volume and variety, the analysis of such data has become a critical challenge as well as a topic of interest. Consequently, statistical methods dealing with the problems associated with these newly developed techniques are in high demand. This special issue of *Genes*, titled *Statistical Methods for the Analysis of Genomic Data*, consists of a number of studies which highlight the state-of-the-art statistical methods for the analysis of genomic data and explore future directions for improvement.

Gene expression is one of the most widely studied topics in genomics. From microarray [1] to high-throughput sequencing of transcriptomes (RNA-Seq) [2], expression levels of tens of thousands of genes can be measured simultaneously. After such data are collected, the first analysis is often to identify genes whose expression levels are associated with experimental conditions or outcomes. Depending on the type of variables, the initial analysis can be done using two-group comparisons (a.k.a. differential expression), linear or Cox regressions, or more complicated statistical models. In clinical studies, the statistical power to identify biologically relevant genes is often limited by the scarce patient samples, which is especially the case for rare diseases such as cancers. Integrated analysis can help improve statistical power by borrowing information across multiple datasets. In [3], Wang et al., introduce a novel penalized regression-based approach for the integrated analysis of gene expression data with survival outcomes. Novel shrinkage penalty functions are proposed to promote similarity among estimated coefficients from each cancer, and the coordinate descent (CD) algorithm is used for model fitting. The proposed method is applied to gene expression data measured using RNA-Seq from The Cancer Genome Atlas (TCGA) project [4] on nine different cancers, and identifies potentially informative genes that are prognostic for patient survival times in multiple cancers.

Due to the large number of genes in a typical genome (e.g., ~25,000 protein coding genes in the human genome), the initial differential expression analysis often identifies many potentially informative genes. To further understand the underlying biology, unsupervised clustering analysis is often conducted to group genes with similar expression patterns together. In the current standard practice, the estimation errors in the gene fold-changes during the initial differential expression analysis are often ignored in the downstream clustering analysis. To address this problem, in [5], Zhang and Di present a novel clustering approach, named MCLUST-ME, which takes the estimation errors in the gene fold-changes into consideration. The proposed model combines the conventional Gaussian mixture clustering model in MCLUST [6] with a random Gaussian measurement error assuming a known variance for each observation, and uses an extended Expectation–Maximization (EM) algorithm for model fitting. A unique feature of MCLUST-ME is that the classification boundary depends on the distribution of the measurement error for each observation, which is shown to achieve improved clustering performance in an RNA-Seq dataset on *Arabidopsis thaliana*.

The analysis of cancer genomic data has long suffered the curse of dimensionality, as sample sizes for most cancer genomic studies are a few hundred at most, while tens of thousands of genomic features are studied. To leverage prior biological knowledge, such as pathways, and more effectively analyze cancer genomic data, the research article by Zeng et al., [7] proposes a Pathway-based Kernel Boosting (PKB) method for integrating gene pathway information for sample classification; the authors use kernel functions calculated from each pathway as base learners and learn the weights through an iterative optimization of the classification loss function. Instead of the first-order approximation used in the usual gradient descent boosting method, used by Wei and Li [8] and Luan and Li [9], the PKB approach uses the second-order approximation of the loss function, which allows for deeper descent at each step. Moreover, the PKB includes two types of regularizations (L1 and L2) for the selection of base learners in each iteration and outperforms other methods, identifying pathways relevant to the outcome variables. The proposed method is applied to gene expression datasets on three cancer types, including breast cancer, melanoma, and glioma, and outperforms competing methods in terms of the prediction of clinical features including tumor grade, tumor site, and metastasis status, as well as the identification of relevant gene pathways.

To study the different roles of the cell cycle pathway in the two subtypes of breast cancer, including luminal A subtype and basal-like subtype using a TCGA (The Cancer Genome Atlas) gene expression dataset, Zhang [10] considers a computational pipeline of detecting differential substructure between two nonparanormal graphical models with false discovery rate control. The proposed approach extends the hierarchical testing method introduced by Liu [11] to a more flexible semiparametric framework and provides a convenient tool for modeling the dependency structure between non-Gaussian data while maintaining the good interpretability and computational convenience of Gaussian graphical models.

Besides transcriptomics, epigenomics has also undergone rapid development in recent years, which provides complementary information for studying cellular functions on top of transcriptomics. Detecting differentially methylated regions (DMRs) based on reduced representation bisulfite sequencing (RRBS) has been widely employed for identifying regions in the genome where the methylation status is associated with the phenotype of interest [12]. Till now, existing methods have been mostly focused on binary phenotypes. Dunbar et al. [13] developed a novel Bayes Factor Method (BFM) to detect genomic loci that are associated with ordinal group responses. Mixed-effect modeling is used to accommodate the correlated methylation states among neighboring CpG (5’—C—phosphate—G—3’) sites. The proposed method is applied to bisulfite sequencing data from a chronic lymphocytic leukemia (CLL) study.

Enhancer-promoter interactions (EPIs) give important information for understanding transcriptional regulation inside cells. However, experimentational approaches investigating EPIs, such as Hi-C [14], are laborious and expensive. Recently, using existing genomic data and machine learning methods to predict EPIs has shown promising results. Xiao et al. [15] have conducted a rigorous study comparing various machine learning methods including convolutional neural networks (CNNs), feed-forward neural networks (FNNs), and gradient boosting with local sequence and 22 epigenomic data types from the K562 cell line on their predictive powers for Epos By randomly splitting the chromosomes rather than the enhancer-promoter pairs, duplication and overlapping cases between training and testing sets are avoided. As a result, they found that local epigenomic features are more predictive of EPIs than local sequences, and combining the two does not provide much predictive gain.

Last but not least, Zhou et al. [16] has developed a novel penalized variable selection method to identify important lipid—environment interactions in a longitudinal lipidomics study. Lipid species play key roles in many biological processes such as signal transduction, cell homeostasis, and energy storage. The authors propose an efficient Newton-Raphson-based algorithm within the generalized estimating equation (GEE) framework. Compared with existing penalization methods [17,18,19,20] in longitudinal studies that have been mostly developed for the identification of important main effects only, the proposed procedure simultaneously selects individual main effect and group structure corresponding to the main lipid effect and interaction effect respectively. The proposed method is applied to a high-dimensional longitudinal lipid dataset from 60 female CD-1 mice in four different treatment groups and identifies markers that show potential association with body weight.

Biologists and statisticians do not always speak the same language, but when they do, the interplay and synergy between them can dramatically advance science. In the modern genomic era, we hope this special issue showcases in a timely manner how novel statistical methods can help improve genomic data analysis, and vice versa, how new challenges in genomic data analysis can inspire method development in statistics.
